# *In vitro* Interactions of Chicken Programmed Cell Death 1 (PD-1) and PD-1 Ligand-1 (PD-L1)

**DOI:** 10.3389/fcimb.2019.00436

**Published:** 2019-12-19

**Authors:** Vishwanatha R. A. P. Reddy, William Mwangi, Yashar Sadigh, Venugopal Nair

**Affiliations:** Viral Oncogenesis Group, The Pirbright Institute, Pirbright, United Kingdom

**Keywords:** chPD-1, chPD-L1, monoclonal antibodies, cancer, pathways

## Abstract

In the present study, we determined the *in vitro* characteristics and binding interactions of chicken PD-1 (chPD-1) and PD-L1 (chPD-L1) and developed a panel of specific monoclonal antibodies against the two proteins. ChPD-1 and chPD-L1 sequence identities and similarities were lower compared with those of humans and other mammalian species. Furthermore, in phylogenetic analysis, chPD-1 and chPD-L1 were grouped separately from the mammalian PD-1 and PD-L1 sequences. As in other species, chPD-1 and chPD-L1 sequences showed signal peptide, extracellular domain, a transmembrane domain and intracellular domain. Based on the three dimensional (3D) structural homology, chPD-1, and chPD-L1 were similar to 3D structures of mammalian PD-1 and PD-L1. Further, Ig V domain of chPD-1 and the Ig V and Ig C domains of chPD-L1 were highly conserved with the mammalian counterparts. *In vitro* binding interaction studies using Superparamagnetic Dynabeads® confirmed that recombinant soluble chPD-1/PD-L1 fusion proteins and surface chPD-1/PD-L1 proteins interacted with each other on COS cells. Two monoclonal antibodies specific against chPD-1 and five antibodies against chPD-L1 were developed and their specific binding characteristics confirmed by immunofluorescence staining and Western blotting.

## Introduction

Programmed death 1 (PD-1; CD279) is a co inhibitory immunoreceptor molecule belonging to the CD28/CTLA-4/ICOS/B7 immunoglobulin (Ig) superfamily (Ishida et al., 1992; Zhang et al., 2004). PD-1 is expressed on activated T cells, B cells, NKT cells, and myeloid cells (Freeman et al., 2006). PD-1 interacts with its specific ligand, Programmed death ligand 1 (PD-L1; CD274), which delivers inhibitory signal that leads to suspension of immune response by inducing apoptosis, anergy, unresponsiveness, and functional exhaustion of T cells (Freeman et al., 2006; Shi et al., 2011). PD-L1 is broadly expressed on both professional and non-professional antigen presenting cells (APCs), and lymphoid and non-lymphoid tissues (Greenwald et al., 2005; Okazaki and Honjo, 2006).

Immune T cell exhaustion is a state of ineffective T cell response that occurs during chronic (latent) viral infections and cancer (Freeman et al., 2006; Wherry, 2011). PD-1 and PD-1/PD-L1 pathways play a key role in the T cell exhaustion (Freeman et al., 2006; Nakamoto et al., 2009; Wherry, 2011). Recent studies have shown that targeting the PD-1 and PD-L1 pathways and PD-1/PD-L1 checkpoints with monoclonal antibodies are promising to reverse the exhausted T-cell response in chronic viral infections and in the treatment of various types of cancer (Blackburn et al., 2009; Nakamoto et al., 2009; Brahmer et al., 2012; Topalian et al., 2012).

In veterinary virology or cancer research, there are a few reports on the PD-1 and PD-L1 homologs in cats, pigs and bovines, however their interactions and potential applications for immunotherapy have not been studied in detail (Jeon et al., 2007; Ikebuchi et al., 2011; Maekawa et al., 2014; Zhu et al., 2017). In the avian species, although there is one report on the chicken homologs chPD-1 and chPD-L1 (Matsuyama-Kato et al., 2012), detailed studies on their biological characteristics and interactions have not been carried out. Moreover, no specific monoclonal antibodies (mAbs) are available against chPD-1 and chPD-L1. In the present study, we describe the molecular characterization of chPD-1/chPD-L1 and their specific interactions, as well as report on the successful generation of specific mAbs that will be valuable in examining future roles in chronic infections and cancer in chickens.

## Materials and Methods

### Cell Culture and Transfection

Monkey kidney cell-derived COS cells and chicken fibroblast cell-derived DF-1 cell were used for the *in vitro* characterization of chPD-1 and chPD-L1. COS and DF-1 cells are well-characterized mammalian and chicken cell lines, respectively. COS and DF-1 cells were grown in high glucose Dulbecco's Modified Eagles Medium (DMEM) with Glutamax, supplemented with 10% fetal calf serum, 1,000 U/ml penicillin and 1 mg/ml streptomycin. DF-1 cells are continuous cell lines of EV-0 chicken embryo fibroblasts. COS and DF-1 cells were transfected by using Lipofectamine 2000 reagent (Invitrogen, Karlsruhe, Germany). COS cells were used for immunofluorescence and Western blot assays. DF-1 cells were used for immunofluorescence staining.

### Characteristics of chPD-1 and chPD-L1

The mammalian orthologs of PD-1 and PD-L1 sequences were retrieved from the NCBI database and multiple sequence alignments were performed using MEGA 6.06. The amino acid sequences of chPD-1 and chPD-L1 were submitted to the Iterative Threading ASSEmbly Refinement (I-TASSER) database online server (https://zhanglab.ccmb.med.umich.edu/I-TASSER/) to identify the predictive 3D structural models (Yang et al., 2015). Further, the 3D models accuracy was predicted in the ModFOLD 6 (http://www.reading.ac.uk/bioinf/ModFOLD/ModFOLD6_form.html) model quality assessment server. The multiple aligned sequences were submitted to ESPript 3.0 (http://espript.ibcp.fr/ESPript/ESPript/index.php) analysis, using I-TASSER database server (https://zhanglab.ccmb.med.umich.edu/I-TASSER/) generated chPD-1 and chPD-L1 protein structures (PDB) as the templates to predict secondary structures of chPD-1 and chPD-L1 (Robert and Gouet, 2014).

### Construction of Expression Constructs of chPD-1 and chPD-L1

The full-length cDNA of chPD-1 and chPD-L1 were amplified by PCR using primers designed from the predicted sequences of the genes in the NCBI databases (Supplementary Table 1). Plasmid pKW06 was used to construct the full length chicken PD1 and PDL1. For total RNA extraction, concanavalin A (Con A) (Sigma-Aldrich, Poole, UK)-stimulated chicken splenocytes were prepared, essentially as described previously (Kaspers et al., 1994). Then, total RNA was extracted using RNeasy mini kit (QIAGEN, Crawley, UK), according to the manufacturer's instructions. First strand synthesis used Superscript III (Invitrogen). After denaturation of the reverse transcriptase at 70 °C for 15 min, 1 μl of the reaction was used in a 50 μl volume polymerase chain reaction (PCR) containing 1 μM dNTP, 10 μM of each primer and 0.625 U of Taq DNA polymerase (Invitrogen). Each cDNA of chPD-1 and chPD-L1 were cloned into pGEM-T vector and sequences confirmed from three independent clones on each strand.

To obtain soluble forms of chPD-1 and chPD-L1, their extracellular domains were identified from comparison of their human and mouse orthologs using the SMART prediction program (http://smart.embl-heidelberg.de/). Primers containing restriction enzyme sites were designed to flank the extracellular domains of chPD-1 and chPD-L1 (Supplementary Table 1). The extracellular domains of chPD-1 and chPD-L1, were amplified and cloned initially into the NhoI and NheI sites of pGEM-T vector (Promega, Southampton, UK), and subsequently subcloned into pKW06 (Staines et al., 2013), to generate the chPD-1-human-IgG1Fc and chPD-L1-human-IgG1Fc fusion constructs to produce the soluble COOH-human Fc-tagged recombinant proteins. All the cloning steps were confirmed by sequence analysis. Surface expression recombinant constructs pKW06-chPD-1 and pKW06-chPD-L1 containing the full-length genes of chPD-1 and chPD-L1, respectively, were also generated.

### Generation of Monoclonal Antibodies Against chPD-1 and chPD-L1

Monoclonal antibodies were produced against the recombinant chPD-1 and chPD-L1. Immunizations and generation of hybridomas were carried out by the DC biosciences, Dundee, Scotland (https://www.dcbiosciences.com/). Hybridomas that cross react with chPD-1 and chPD-L1 were initially selected based on dot blot ELISA (data not shown).

### Immunofluorescence Staining

COS and DF-1 cells (1 × 10^6^) were plated in a six-well culture plates. Recombinant expression constructs pKW06-chPD-1, pKW06-chPD-L1, pKW06-chPD-1-human-IgG1Fc, and pKW06-chPD-L1-human-IgG1Fc were transfected into COS cells (90–95% confluence) by Lipofectamine 2000 (Invitrogen, Karlsruhe, Germany). COS cells were fixed in 4% paraformaldehyde [30 min, Room temperature (RT)] and permeabilized with 0.1% Triton X-100 (15 min, RT). After blocking in 5% bovine serum albumin (BSA) in phosphate-buffered saline (PBS) for 30 min, cells were incubated (1 h, 37°C) with mouse monoclonal anti-chPD-1 and chPD-L1 antibodies (1:500 in 5% BSA). After washing the cells three times to remove any unbound antibodies, cells were incubated (1 h, 37°C) with Alexa 568 (COS cells)/488 (DF-1 cells)-conjugated goat anti-mouse antibodies (1:200 in 5% BSA). Finally, after washing, cells were stained (10 min, RT) with DAPI (1:10000) and viewed by using a Leica (Wetzlar, Germany) TCS SP2 confocal laser-scanning microscope. The experiments were performed in triplicate.

### Western Blot Analysis

The supernatants from COS cells transfected with pKW06-chPD-1-human-IgG1Fc and pKW06-chPD-L1-human-IgG1Fc were treated with TruPAGE LDS sample buffer and boiled for 10 min at 95°C. The samples were then loaded on a 4–12% TruPAGE Precast Gel (Bio-Rad) and transferred onto PVDF membranes. Immunoblots were blocked with 5% skim milk (2.5 g skim milk powder was dissolved in 50 ml of TBST) for 1 h at room temperature. Soluble proteins of chPD-1 and chPD-L1 were detected using monoclonal anti-chPD-1 and chPD-L1 antibodies (1:50 in 5% skim milk), after which blots were washed three times in TBST. Then, blots were incubated (1 h, 37°C) with secondary antibody IRDye 680RD goat anti-mouse IgG (1:200 in 5% skim milk). Finally, after washing, blots were visualized using Odyssey Clx (LI-COR).

### Binding of Soluble chPD-1 and chPD-L1 Coated Dynabeads® to COS Cells Expressing Respective Ligands

Dynabeads® Biotin Binder are 2.8 μm superparamagnetic beads that binds biotinylated ligands (proteins or antibodies) were used to examine the interactions between chPD-1 and chPD-L1. Dynabeads® Biotin Binder (45 μl) were mixed with 7.5 μg/ml of biotinylated rabbit anti-human IgG, in 1,500 μl of 0.05% PBS-Tween, and incubated on a rotary mixer (1 h, RT). After 1 h incubation, the tube was held against a magnet to attract the conjugated beads and the fluid was removed using a pipette. The conjugated beads were then washed twice with PBS-Tween by magnet as described above. Soluble chPD-1-human-IgG1Fc and chPD-L1-human-IgG1Fc fusion proteins of an estimated concentration of 5 μg/ml were incubated with the Dynabeads on rotary mixer (1 h, RT) and washed two times with PBS-T and one time with PBS. Dynabead-bound fusion proteins were suspended in 500 μl of culture medium containing FCS, and incubated with COS cells transfected with pKW06-chPD-1 and pKW06-chPD-L1 plasmids (1 h, 37°C). Cells were washed and fixed with 1:1 acetone: methanol and stained with Giemsa (2 min, RT). Before imaging on the EVOS digital microscope (ThermoFisher Scientific, USA). The percentage of binding of surface expressed chPD-1 to soluble chPD-L1-human-IgG1Fc and surface expressed chPD-L1 to soluble chPD-1-human-IgG1Fc was determined in 10 randomly selected fields by scoring COS cells with 5 or more beads as positive under Leica (Wetzlar, Germany) DM IRB light microscope. IF staining and confocal microscopy analysis were also performed by using anti-chPD-1 and chPD-L1 monoclonal antibodies as described above.

## Results

### Cloning and Analysis of the chPD-1 and chPD-L1

**Figure 1:**
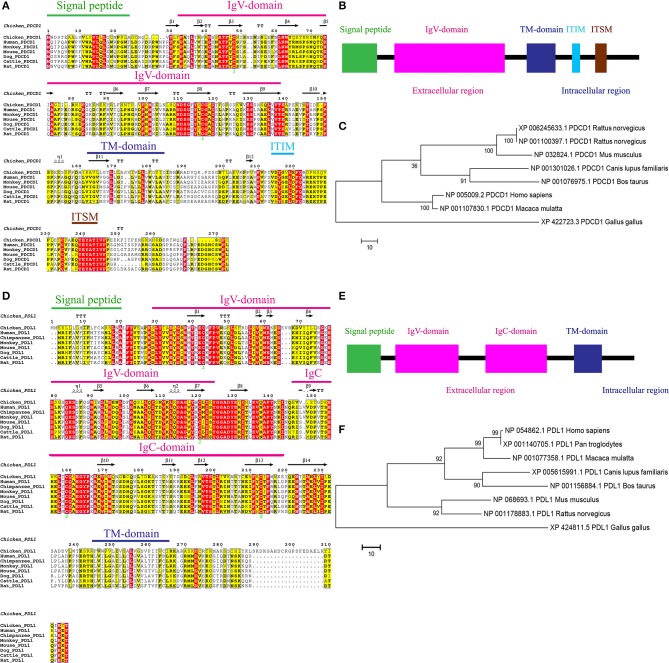
Alignment of the amino acid sequences of chPD-1 and chPD-L1 with their mammalian orthologs. **(A,D)** Qualitative analysis of sequence identity and similarity using the ESPript 3.0 online tool. The predicted secondary structures are marked above the alignment (by helices with squiggles, β strands with arrows, and turns with TT letters) and are based on the PD-1 and PD-L1 structural models. Strictly conserved residues are boxed in white on a red background, more conserved residues are boxed in black on a yellow background, and less conserved residues are boxed in black on a white background. Shaded areas represent conservation of amino acid, the darker the shading, the more conserved the residue across species. The green number in Ig V domain of chPD-1 and Ig V and Ig C domains of chPD-L1 indicate that the two cysteine residues that form an intrachain disulfide bridge, respectively. **(B,E)** Predicted functional motifs in chPD-1 contains extracellular, transmembrane and intracellular domains and chPD-L1 contains extracellular, transmembrane and intracellular domains. **(C,F)** Maximum likelihood phylogenetic trees based on amino acid sequences of chPD-1 and chPD-L1 in relation to other animal species. Bootstrap values of 1,000 replicates was assigned for the analysis.

To clone chPD-1 and chPD-L1 gene, total RNA was extracted from Con A-stimulated chicken splenocytes, essentially as described previously (Kaspers et al., 1994), and cDNA synthesized. The ORFs of chPD-1 and chPD-L1 was found to be 273 and 315 amino acids in length, respectively (Figures 1A,D). The mammalian orthologs of PD-1 and PD-L1 sequences were retrieved from the NCBI database and multiple sequence alignments were performed. ESPript 3.0 server was used to build identities and similarities among the orthologs. ChPD-1 sequences identities and similarities, respectively, with human (30.76%; 39.56%), cattle (29.3%; 36.66%), mouse (27.83%; 36.99%), dog (27.47%; 36.63%), and rat (27.1%; 35.89%), were relatively lower when compared among mammalian species (Supplementary Tables 2, 3). Whereas, chPD-L1 sequences identities and similarities, respectively, with human (38.62%; 50.68%), cattle (36.66%; 47.05%), mouse (38.62%; 48.96%), dog (37.71%; 48.44%), and rat (39.65%; 50.34%) were slightly higher (Supplementary Tables 2, 3).

ChPD-1 and chPD-L1 sequences contained the signal peptide, extracellular domain, a transmembrane domain, and intracellular domain, similar to other species (Figures 1B,E). PD-1 has composed of an Ig Variable type (V-type) domain in extracellular N-terminal domain between positions 31 to 139 amino acids. The cysteine residues at 48 and 116 that form the intrachain disulfide bond to construct Ig V domain of PD-1, were highly conserved among all the ortholog species. In the Ig V domain of PD-1, ATF (44–46), LNW (61–63), NDSG (109–112), ES (129 and 130), and VTE (137–139) sequences were highly conserved (Figure 1A). The intracellular C-terminal PD-1 domain contains a well-conserved two tyrosine (Y) residues, one in an immunoreceptor tyrosine-based inhibitory motif (ITIM: S/I/L/VxYxxL/V) and another in immunoreceptor tyrosine-based switch motif (ITSM: TxYxxL) (Figure 1A). The amino acid sequence TEYATIVF around the C-terminal tyrosine is also highly conserved among all the species. PD-L1 is composed of an Ig V-type domain between 29 and 124 amino acids and Ig constant-type (C-type) domain between 148 and 219 amino acids, in extracellular domain (Figure 1D). The cysteine residues form the intrachain disulfide bond at positions 43 and 120 to construct Ig V domain and at positions 133 and 225 to construct Ig C domain of PD-L1, were also highly conserved among all the ortholog species. In the IgV domain of PD-1, ATF (44–46), LNW (61–63), NDSG (109–112), ES (129 and 130), and VTE (137–139) sequences were highly conserved (Figure 1A). Phylogenetic analysis showed that chPD-1 and chPD-L1 were grouped separately from the mammalian PD-1 and PD-L1 sequences (Figures 1C,F).

### Homology Models of chPD-1 and chPD-L1

**Figure 2:**
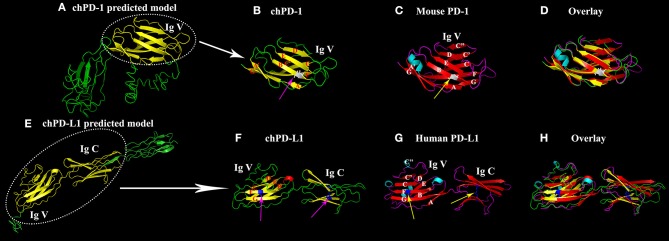
Similarity of the Ig V domain of chPD-1 to the Ig V domain of mouse PD-1 and the Ig V and Ig C domains of chPD-L1 to the Ig V and Ig C domains of human PD-L1. **(A,E)** Predicted homology structural models of chPD-1 and chPD-L1 by I-TASSER software. Although difference in the number of presence of β sheets, the overall structures have identical orientations. **(B–D)** Overlay of Ig V domains of chPD-1 with Ig V mouse PD-1. **(B)** The Ig V domain of chPD-1 is shown in yellow, and **(C)** the Ig V domain mouse PD-1 is shown in red. In mouse PD-1, the two β-sheets are labeled A'GFCC'C” and ABED. In chPD-1, C” and C-terminal of G are absent. The cysteine residues at 48 and 116 form intrachain disulfide bonds are drawn as balls and sticks, and are shown in gray (pink arrow for chPD-1 and yellow arrow for mouse PD-1). **(F–H)** Overlay of Ig V and Ig C domains of chPD-L1 with human PD-L1. **(F)** The Ig V and Ig C domains of chPD-L1 is shown in yellow, and the **(G)** Ig V and Ig C domains of human PD-L1 is shown in red. In human PD-L1, the two β-sheets are labeled AGFCC'C” and BED. In chPD-L1, A, C', and C” are absent. The cysteine residues at 43 and 120 of Ig V domain and at 133 and 225 of Ig C domain form intrachain disulfide bonds are drawn as balls and sticks, and are shown in blue (pink arrow for chPD-L1 and yellow arrow for human PD-L1). Both chPD-1 and chPD-L1 were showed in same view, respectively.

The homology 3D models of chPD-1 and chPD-L1 were built by I-TASSER online tool. For chPD-1, the predicted top model 1 was with: Confidence score (C-score): −3.09, TM-score: 0.37 ± 0.12 and root mean square deviation (RMSD): 15.3 ± 3.4 Å (Figure 2A). The best model was used for structural similarity simulation and chPD-1 was found to have highest similarity with the catalytic antibody 28B4, which involved in periodate-dependent oxygenation of sulfide 1 to sulfoxide 2. Furthermore, catalytic antibodies are shown to use for the elucidation of the molecular mechanisms of the immune response and origins of enzymatic catalysis (Yin et al., 2001). For chPD-L1, the predicted top model 1 was with: Confidence score: −1.68, TM-score: 0.51 ± 0.15 and RMSD: 10.1 ± 4.6 Å (Figure 2E). The best model was used for structural similarity simulation and chPD-L1 was found to have highest similarity with the SYG-1 and SYG-2 cell adhesion molecules (CAMs). SYG-1 and SYG-2 CAMs have been reported to play diverse role ranging from function in neural development to formation of kidney filtration barrier (Ozkan et al., 2014). The ModFOLD model quality assessment server 6 was used to assess the quality of the chPD-1 and chPD-L1 3D models, and was CERT confidence with *p* < 0.001 (McGuffin and Roche, 2011; McGuffin et al., 2018).

Cartoon 3D structural diagram of chPD-1 Ig V domain shows a two layer β sandwich, a topology characteristic of Ig V type domains (Figure 2B). A superimposition of Ig V domains of chPD-1 and mouse PD-1 showed a good overlap between the structures (Figures 2B–D). In a two layer β sandwich of Ig V domain, front A'GFCC'C” and back ABED strands are present in mouse PD-1 (Lin et al., 2008), however C” and C-terminal of G β sheets were absent in chPD-1 (Figures 2B,C). Cartoon structural diagram of chPD-L1 IgV and IgC domains reveals a characteristic Ig type topology (Figure 2F). IgV and IgC domains of chicken PD-L1 and human PD-L1 superimposition showed a good overlapping structure (Figures 2F–H). A two layer β sandwich, front AGFCC'C” and back BED strands are characteristic of Ig V domain of human PD-L1 (Lin et al., 2008), but A, C' and C” were absent in chPD-L1 (Figures 2F,G).

### Specificity of the mAbs Raised Against chPD-1 and chPD-L1

**Figure 3:**
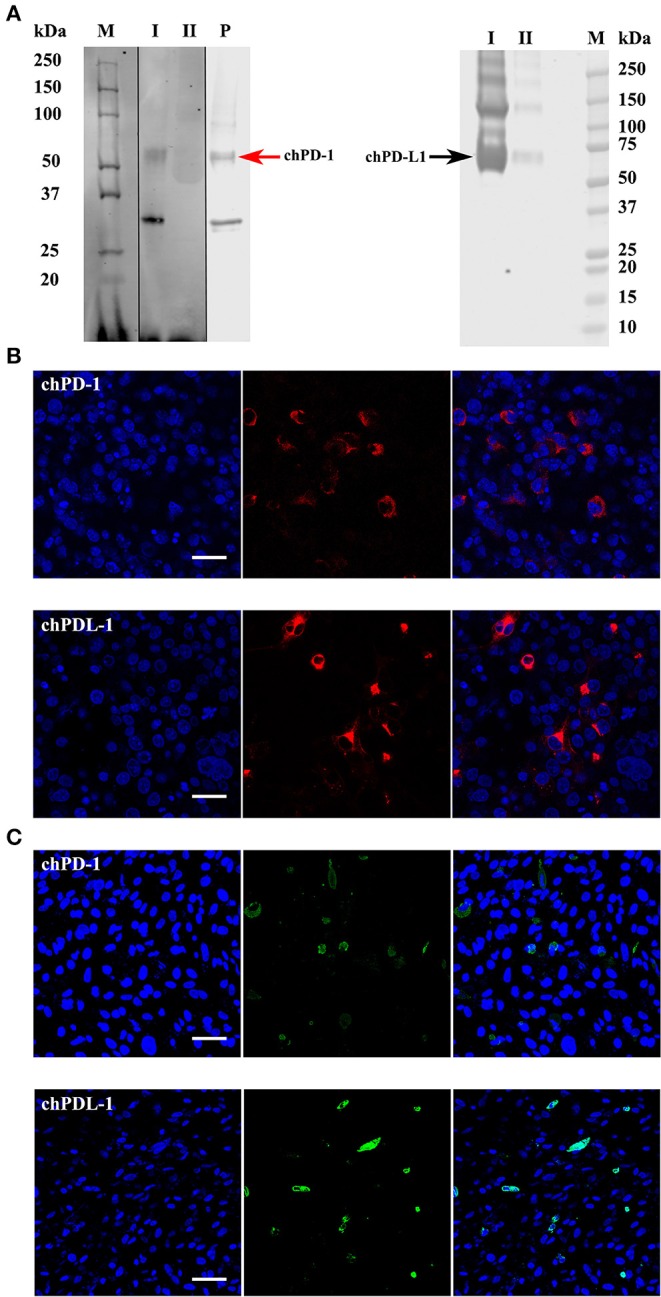
Western blots and confocal micrographs of chPD-1 and chPD-L1. **(A)** The extracellular soluble domains of chPD-1 and chPD-L1 were sub cloned into pkW06 expression vector and expressed as a human Fc-tagged protein, namely, pKW06-chPD-1-human-IgG1Fc and pKW06-chPD-L1-human-IgG1Fc. Proteins were resolved on an SDS 4–12% Bis-Tris gel along with 10% of input protein and western blotted with monoclonal anti-chPD-1 and chPD-L1 antibodies. Images demonstrate that ~55 kDa (red arrow left direction) for chPD-1 and a band of ~70 kDa (black arrow right direction) for chPD-L1. I is purified sample, II is supernatant from transfected cells and P is commercial positive control. Dividing black line indicates that intervening lanes have been spliced out. **(B,C)** Representative confocal micrographs illustrating chPD-1 and chPD-L1 in COS (left) and DF-1 (right) cells. Immunofluorescence staining was carried out on COS and DF-1 cells transfected with expression constructs containing pKW06-chPD-1-human-IgG1Fc and pKW06-chPD-L1-human-IgG1Fc plasmids. Cells were fixed after 24–48 h and stained with monoclonal anti-chPD-1 **(A)** and chPD-L1 **(B)** antibodies. Scale bar represents 40 μm.

Initial dot blot ELISA screening showed that the supernatants from two hybridomas reacted with chPD-1 and five hybridomas reacted with chPD-L1 (data not shown). Western blot analysis confirmed that two chPD-1 antibodies and five chPD-L1 antibodies identified in the dot blot ELISA were specific and identified the respective proteins (Supplementary Table 4). Figure 3A shows a representative Western blot image demonstrating the specific binding of the mAbs to the ~55 kDa chPD-1 and the ~70 kDa chPD-L1, respectively. Figure 3B is a representative image of immunofluorescence staining of COS cells transfected with pKW06-chPD-1-human-IgG1Fc and pKW06-chPD-L1-human-IgG1Fc plasmids using the specific mAbs (see Supplementary Files for individual figures of different hybridomas or their clones). Furthermore, we have confirmed that both the PD-1 and PD-L1 antibodies were specific for chPD-1 and chPD-L1 in chicken DF-1 cells (Figure 3C).

### chPD-1 and chPD-L1-Specific mAbs Do Not Block the Interaction of the Two Proteins

**Figure 4:**
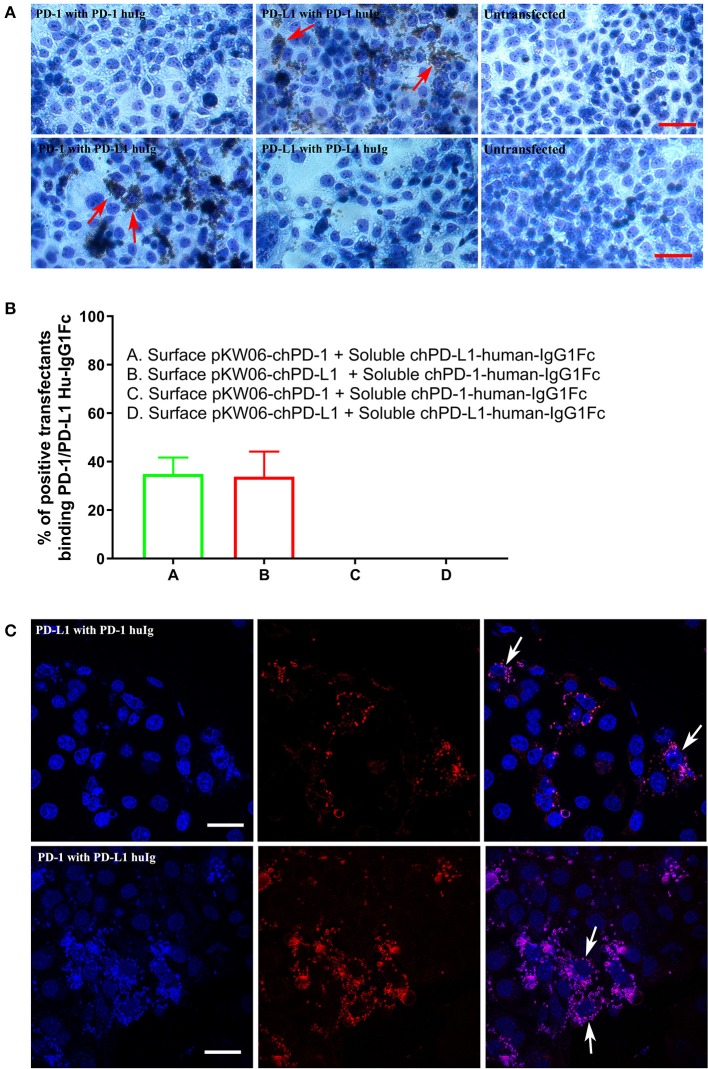
Binding interactions of recombinant soluble chPD-1/PD-L1 with surface chPD-1/PD-L1 on COS cells. **(A)** Representative EVOS images illustrating chPD-1 and chPD-L1 interactions. Images were taken using an EVOS digital microscope. Giemsa staining was carried out on COS cells transfected with surface expression constructs pKW06-chPD-1and pKW06-chPD-L1. Rosette formation was observed when surface pKW06-chPD-L1 interacts with soluble chPD-1-human-IgG1Fc fusion protein (red arrows) and surface pKW06-chPD-1 with soluble chPD-L1-human-IgG1Fc fusion protein (red arrows). No rosettes was observed in surface pKW06-chPD-1 with soluble chPD-1-human-IgG1Fc fusion protein interaction, surface pKW06-chPD-L1 with soluble chPD-L1-human-IgG1Fc fusion protein interaction, and untransfected cells. Scale bar represents 50 μm. **(B)** Percentage of soluble chPD-1/PD-L1-human-IgG1Fc fusion proteins interactions with surface pKW06-chPD-1/PD-L1 were calculated by counting 10 randomly selected fields by scoring COS cells with 5 or more beads as positive. On surface pKW06-chPD-1 and pKW06-chPD-L1 transfected COS cells, 34.89 ± 6.8% of chPD-L1-human-IgG1Fc and 33.79 ± 10.3% of chPD-1-human-IgG1Fc showed clear rosette structures, respectively. Data are represented as means of three independent biological experiments ± standard deviation (error bars). **(C)** Representative confocal photo micrographs illustrating clear rosette (white arrows) formation during chPD-1 and chPD-L1 interactions. Red fluorescence visualizes Dynabeads®. Scale bar represents 40 μm.

For the PD-1 and PD-L1 binding assay, the soluble chPD-1-human-IgG1Fc and chPD-L1-human-IgG1Fc fusion proteins were immobilized onto Dynabeads® by using human IgG and incubated on COS cells transfected with the pKW06-chPD-1 and pKW06-chPD-L1 plasmids. After fixation and staining, specific binding was quantified by counting the beads under light microscope. The soluble chPD-1-human-IgG1Fc fusion protein was bound to surface of the pKW06-chPD-L1 transfected COS cells, as observed by rosette formation (Figure 4A). Similarly, the soluble chPD-L1-human-IgG1Fc fusion protein was bound to the surface of pKW06-chPD-1 transfected cells with clear rosette formation (Figure 4A). It was estimated that 34.89 ± 6.8% of chPD-L1-human-IgG1Fc fusion proteins and 33.79 ± 10.3% of chPD-1-human-IgG1Fc fusion proteins showed clear rosette interaction on surface pKW06-chPD-1 and pKW06-chPD-L1 transfected COS cells (Figure 4B). The presence of clear rosette between chPD-1 and chPD-L1 was confirmed also by IF staining (Figure 4C). Using this binding assay, we examined whether any of the PD-1 or PD-L1-specific mAbs developed in this study interfered with the specific interactions of PD-1 and PD-L1. Despite the ability of the mAbs to bind to the two proteins, they were unable to inhibit or reduce the interaction of chPD-1 and chPD-L1 binding interactions.

## Discussion

The immune system plays an important role in the control of viral pathogenesis and tumorigenesis (Cully, 2017; Hashimoto et al., 2018). During chronic viral infections the increased level or duration of stimulation of virus specific CD8 T cells leads to non-functional state called T cell exhaustion (Freeman et al., 2006). Recent studies have shown that PD1 is highly expressed on exhausted T cells, and PD-1/PD-L1 checkpoints are targets of immunotherapy. Currently, in humans, usage of monoclonal antibodies as blockades of PD-1 and PD-L1 pathways, and inhibitors of the PD-1/PD-L1 interaction are gaining momentum (Brahmer et al., 2012; Topalian et al., 2012). However, chPD-1 and chPD-L1 have not been characterized and no specific mAbs have been developed. We have now characterized the chicken PD-1 and PD-L1, studied their binding interactions and developed specific mAbs against chPD-1 and chPD-L1.

The PD-1 and PD-L1 sequence homologies were lower in chPD-1 and chPD-L1 sequences compared with those of the mammalian species (Jeon et al., 2007; Gjetting et al., 2019). The conservation of the ITIM and ITSM motifs in the C-terminal, and highly conserved TEYATIVF indicated that the immune signal regulation associated with PD-1 is highly conserved among species (Okazaki et al., 2001). The stoichiometry between human PD-1 and PD-L1 reported to form a 1:1 complex, chickens may also form similar ratio because the predicted 3D structures of the domains suggested identical configurations (Lin et al., 2008). C-score estimates the quality of predicted models, and −3.09 of chPD-1 and −1.68 of chPD-L1 signifies that both were within the usual confidence range of −5 to 2 (Yang et al., 2015). TM-score and RMSD give information on structural similarity with other structures, and correlation with C-score.

In the present study, we showed that recombinant chPD-1 and chPD-L1 interacted with each other. The strong binding interactions between chPD-1 and chPD-L1 demonstrated that these molecules participate in the suppression of antigen specific immune responses, which is similar to other species (Okazaki et al., 2001; Carter et al., 2002; Maekawa et al., 2014). Furthermore, the development of specific mAbs against the chicken homologs gives the potential for inhibiting the PD-1/PD-L1 interaction pathways for immunotherapy in chronic diseases in avian species. However, non-inhibition of chPD-1 and chPD-L1 interactions by the panel of mAbs developed in this study suggested that the specific antigenic epitopes identified by these mAbs are most likely outside the interacting domains of the PD-1 and PD-L1. Further detailed structural and mutagenesis studies are needed to identify the epitopes of these specific mAbs to understand why they are unable to interfere with the PD-1/PD-L1 interactions.

In conclusion, we describe the characterization of the chPD-1 and chPD-L1 molecules and their interactions. We have also developed a panel of mAbs that specifically identified the chicken PD-1 and PD-L1 homologs. Despite their specific binding to the chPD-1 and chPD-L1, none of the mAbs were able to prevent the interactions of the two proteins. Nevertheless, the results of this work will form the technical basis for future research to explore the role of PD-1/PD-L1 pathway in the latency mechanisms and immunosuppression of Marek's disease and other chronic viral infections of chickens.

## Data Availability Statement

All datasets generated for this study are included in the article/Supplementary Material.

## Author Contributions

VR, WM, and VN conceived and designed the study, analyzed the data, and drafted the manuscript. VR and WM performed the experiments. YS participated in design and analysis of the results. All authors read and approved the final manuscript.

### Conflict of Interest

The authors declare that the research was conducted in the absence of any commercial or financial relationships that could be construed as a potential conflict of interest.

## Abbreviations

chPD-1: Chicken Programmed death 1
chPD-L1: Chicken Programmed death ligand 1
RT-qPCR: Real time quantitative PCR.

## References

[B1] Blackburn S. D. Shin H. Haining W. N. Zou T. Workman C. J. Polley A.. (2009). Coregulation of CD8+ T cell exhaustion by multiple inhibitory receptors during chronic viral infection. Nat. Immunol. 10, 29–37. 10.1038/ni.1679 19043418 PMC2605166

[B2] Brahmer J. R. Tykodi S. S. Chow L. Q. Hwu W. J. Topalian S. L. Hwu P.. (2012). Safety and activity of anti-PD-L1 antibody in patients with advanced cancer. N. Engl. J. Med. 366, 2455–2465. 10.1056/NEJMoa1200694 22658128 PMC3563263

[B3] Carter L. Fouser L. A. Jussif J. Fitz L. Deng B. Wood C. R.. (2002). PD-1:PD-L inhibitory pathway affects both CD4(+) and CD8(+) T cells and is overcome by IL-2. Eur. J. Immunol. 32, 634–643. 10.1002/1521-4141(200203)32:3<634::AID-IMMU634>3.0.CO;2-9 11857337

[B4] Cully M. (2017). Viral infections: reinvigorating exhausted T cells in hepatitis B infection. Nat. Rev. Drug Discov. 16:240. 10.1038/nrd.2017.48 28356588

[B5] Freeman G. J. Wherry E. J. Ahmed R. Sharpe A. H. (2006). Reinvigorating exhausted HIV-specific T cells via PD-1-PD-1 ligand blockade. J. Exp. Med. 203, 2223–2227. 10.1084/jem.20061800 17000870 PMC2118103

[B6] Gjetting T. Gad M. Frohlich C. Lindsted T. Melander M. C. Bhatia V. K.. (2019). Sym021, a promising anti-PD1 clinical candidate antibody derived from a new chicken antibody discovery platform. MAbs 11, 666–680. 10.1080/19420862.2019.1596514 31046547 PMC6601539

[B7] Greenwald R. J. Freeman G. J. Sharpe A. H. (2005). The B7 family revisited. Annu. Rev. Immunol. 23, 515–548. 10.1146/annurev.immunol.23.021704.115611 15771580

[B8] Hashimoto M. Kamphorst A. O. Im S. J. Kissick H. T. Pillai R. N. Ramalingam S. S.. (2018). CD8 T cell exhaustion in chronic infection and cancer: opportunities for interventions. Annu. Rev. Med. 69, 301–318. 10.1146/annurev-med-012017-043208 29414259

[B9] Ikebuchi R. Konnai S. Shirai T. Sunden Y. Murata S. Onuma M.. (2011). Increase of cells expressing PD-L1 in bovine leukemia virus infection and enhancement of anti-viral immune responses *in vitro* via PD-L1 blockade. Vet. Res. 42:103. 10.1186/1297-9716-42-103 21943148 PMC3195098

[B10] Ishida Y. Agata Y. Shibahara K. Honjo T. (1992). Induced expression of PD-1, a novel member of the immunoglobulin gene superfamily, upon programmed cell death. EMBO J. 11, 3887–3895. 10.1002/j.1460-2075.1992.tb05481.x 1396582 PMC556898

[B11] Jeon D. H. Oh K. Oh B. C. Nam D. H. Kim C. H. Park H. B.. (2007). Porcine PD-L1: cloning, characterization, and implications during xenotransplantation. Xenotransplantation 14, 236–242. 10.1111/j.1399-3089.2007.00403.x 17489864

[B12] Kaspers B. Lillehoj H. S. Jenkins M. C. Pharr G. T. (1994). Chicken interferon-mediated induction of major histocompatibility complex class II antigens on peripheral blood monocytes. Vet. Immunol. Immunopathol. 44, 71–84. 10.1016/0165-2427(94)90170-8 7536986

[B13] Lin D. Y. Tanaka Y. Iwasaki M. Gittis A. G. Su H. P. Mikami B.. (2008). The PD-1/PD-L1 complex resembles the antigen-binding Fv domains of antibodies and T cell receptors. Proc. Natl. Acad. Sci. U.S.A. 105, 3011–3016. 10.1073/pnas.0712278105 18287011 PMC2268576

[B14] Maekawa N. Konnai S. Ikebuchi R. Okagawa T. Adachi M. Takagi S.. (2014). Expression of PD-L1 on canine tumor cells and enhancement of IFN-gamma production from tumor-infiltrating cells by PD-L1 blockade. PLoS ONE 9:e98415. 10.1371/journal.pone.0098415 24915569 PMC4051644

[B15] Matsuyama-Kato A. Murata S. Isezaki M. Kano R. Takasaki S. Ichii O.. (2012). Molecular characterization of immunoinhibitory factors PD-1/PD-L1 in chickens infected with Marek's disease virus. Virol. J. 9:94. 10.1186/1743-422X-9-94 22612856 PMC3447683

[B16] McGuffin L. J. Roche D. B. (2011). Automated tertiary structure prediction with accurate local model quality assessment using the IntFOLD-TS method. Proteins 79(Suppl. 10), 137–146. 10.1002/prot.23120 22069035

[B17] McGuffin L. J. Shuid A. N. Kempster R. Maghrabi A. H. A. Nealon J. O. Salehe B. R.. (2018). Accurate template-based modeling in CASP12 using the IntFOLD4-TS, ModFOLD6, and ReFOLD methods. Proteins 86 (Suppl. 1), 335–344. 10.1002/prot.25360 28748648

[B18] Nakamoto N. Cho H. Shaked A. Olthoff K. Valiga M. E. Kaminski M.. (2009). Synergistic reversal of intrahepatic HCV-specific CD8 T cell exhaustion by combined PD-1/CTLA-4 blockade. PLoS Pathog. 5:e1000313. 10.1371/journal.ppat.1000313 19247441 PMC2642724

[B19] Okazaki T. Honjo T. (2006). The PD-1-PD-L pathway in immunological tolerance. Trends Immunol. 27, 195–201. 10.1016/j.it.2006.02.001 16500147

[B20] Okazaki T. Maeda A. Nishimura H. Kurosaki T. Honjo T. (2001). PD-1 immunoreceptor inhibits B cell receptor-mediated signaling by recruiting src homology 2-domain-containing tyrosine phosphatase 2 to phosphotyrosine. Proc. Natl. Acad. Sci. U.S.A. 98, 13866–13871. 10.1073/pnas.231486598 11698646 PMC61133

[B21] Ozkan E. Chia P. H. Wang R. R. Goriatcheva N. Borek D. Otwinowski Z.. (2014). Extracellular architecture of the SYG-1/SYG-2 adhesion complex instructs synaptogenesis. Cell 156, 482–494. 10.1016/j.cell.2014.01.004 24485456 PMC3962013

[B22] Robert X. Gouet P. (2014). Deciphering key features in protein structures with the new ENDscript server. Nucleic Acids Res. 42, W320–W324. 10.1093/nar/gku316 24753421 PMC4086106

[B23] Shi F. Shi M. Zeng Z. Qi R. Z. Liu Z. W. Zhang J. Y.. (2011). PD-1 and PD-L1 upregulation promotes CD8(+) T-cell apoptosis and postoperative recurrence in hepatocellular carcinoma patients. Int. J. Cancer 128, 887–896. 10.1002/ijc.25397 20473887

[B24] Staines K. Young J. R. Butter C. (2013). Expression of chicken DEC205 reflects the unique structure and function of the avian immune system. PLoS ONE 8:e51799. 10.1371/journal.pone.0051799 23326318 PMC3541370

[B25] Topalian S. L. Hodi F. S. Brahmer J. R. Gettinger S. N. Smith D. C. McDermott D. F.. (2012). Safety, activity, and immune correlates of anti-PD-1 antibody in cancer. N. Engl. J. Med. 366, 2443–2454. 10.1056/NEJMoa1200690 22658127 PMC3544539

[B26] Wherry E. J. (2011). T cell exhaustion. Nat. Immunol. 12, 492–499. 10.1038/ni.2035 21739672

[B27] Yang J. Yan R. Roy A. Xu D. Poisson J. Zhang Y. (2015). The I-TASSER suite: protein structure and function prediction. Nat. Methods 12, 7–8. 10.1038/nmeth.3213 25549265 PMC4428668

[B28] Yin J. Mundorff E. C. Yang P. L. Wendt K. U. Hanway D. Stevens R. C.. (2001). A comparative analysis of the immunological evolution of antibody 28B4. Biochemistry 40, 10764–10773. 10.1021/bi010536c 11535051

[B29] Zhang X. Schwartz J. C. Guo X. Bhatia S. Cao E. Lorenz M.. (2004). Structural and functional analysis of the costimulatory receptor programmed death-1. Immunity 20, 337–347. 10.1016/S1074-7613(04)00051-2 15030777

[B30] Zhu Y. P. Yue F. He Y. Li P. Yang Y. Han Y. T.. (2017). Prokaryotic expression of the extracellular domain of porcine programmed death 1 (PD-1) and its ligand PD-L1 and identification of the binding with peripheral blood mononuclear cells *in vitro*. Can. J. Vet. Res. 81, 147–154. 28408783 PMC5370541

