# Supplementary material for: In vitro Interactions of Chicken Programmed Cell Death 1 (PD-1) and PD-1 Ligand-1 (PD-L1)
Source: Front Cell Infect Microbiol. 2019 Dec 19;9:436. doi: 10.3389/fcimb.2019.00436 (PMC6930881; doi:10.3389/fcimb.2019.00436)

Supplementary Table 1. List of primers used for construction of recombinant chPD-1 and chPD-L1 plasmids for full length cDNA and extracellular coding region 3 **amplification.**

Full length cDNA amplification	Sequence (5' - 3')
chPD-1 -Forward	ATTGCTAGCGCTCTGGGCACCTCG
chPD-1 -Reverse	ATTGCGGCCGCTAAATGGCCCTCAG
chPD-L1 -Forward	ATTGCTAGCGTTCTACCTACATGATG
chPD-L1 -Reverse	ATTGCGGCCGCCCTGCTTTCTCTAAG
**Extracellular coding amplification**	**Sequence (5' - 3')**
chPD-1 -Forward	ATTGCTAGCGCTCTGGGCACCTCG
chPD-1 -Reverse	TCGAGATCTGGGGGGCTGCTTTCC
chPD-L1 -Forward	ATTGCTAGCGTTCTACCTACATGATG
chPD-L1 -Reverse	TTAAGATCTTCAGTCCAGAGTACGTCATC

Supplementary Table 2. The amino acid sequence identities of PD-1 and PD-L1 among animal species.

Species	Chicken	Human	Monkey	Mouse	Dog	Cattle	Rat
**Chicken**	-	38.62	38.62	38.62	37.71	36.33	39.65
**Human**	30.76	-	92.06	69.65	76.12	73.35	70
**Monkey**	30.76	96.18	-	68.62	74.39	72.66	68.62
**Mouse**	27.83	59.72	60.41	-	67.12	65.74	84.48
**Dog**	27.47	66.31	65.62	55.55	-	78.89	68.51
**Cattle**	29.3	65.6	66.31	51.77	70.92	-	66.78
**Rat**	27.1	61.32	63.76	86.75	57.49	56.73	-

Lower section shows amino acid identity percentage homologies of PD-1, and higher section shows PD-L1.

Supplementary Table 3. The amino acid sequence similarities of PD-1 and PD-L1 among animal species.

Species	Chicken	Human	Monkey	Mouse	Dog	Cattle	Rat
**Chicken**	-	50.68	50	48.96	48.44	47.05	50.34
**Human**	39.56	-	92.41	76.55	77.5	77.85	75.51
**Monkey**	39.56	96.87	-	75.51	76.47	76.81	73.44
**Mouse**	36.99	64.58	64.93	-	73.7	73.7	86.55
**Dog**	36.63	69.79	69.44	60.06	-	81.66	74.04
**Cattle**	36.63	68.79	69.14	56.02	73.04	-	73.35
**Rat**	35.89	66.2	68.29	87.45	62.71	61.7	-

Lower section shows amino acid similarity percentage homologies of PD-1, and higher section shows PD-L1.

Supplementary Table 4. List of monoclonal antibodies specific against chPD-1 and chPD-L1.

Monoclonal antibodies name	Specificity against	Subclass	Isotype
2C10	ChPD-1	IgG1	Kappa
5E2	ChPD-1	IgG3	Kappa
2A6	ChPD-L1	IgG	Kappa
3A2	ChPD-L1	IgG	Kappa
4E4	ChPD-L1	IgG	Kappa
4G7	ChPD-L1	IgG	Kappa
6E5	ChPD-L1	IgG	Kappa

Supplementary Figure 1. Monoclonal antibodies clones, namely, 2C10 and 5E2 specific against chPD-1 and 2A6, 3A2, 4E4, 4G7 and 6E5 specific against chPD-L1. Scale bar represents 60 μm for chPD-1 and 40 μm for chPD-L1.

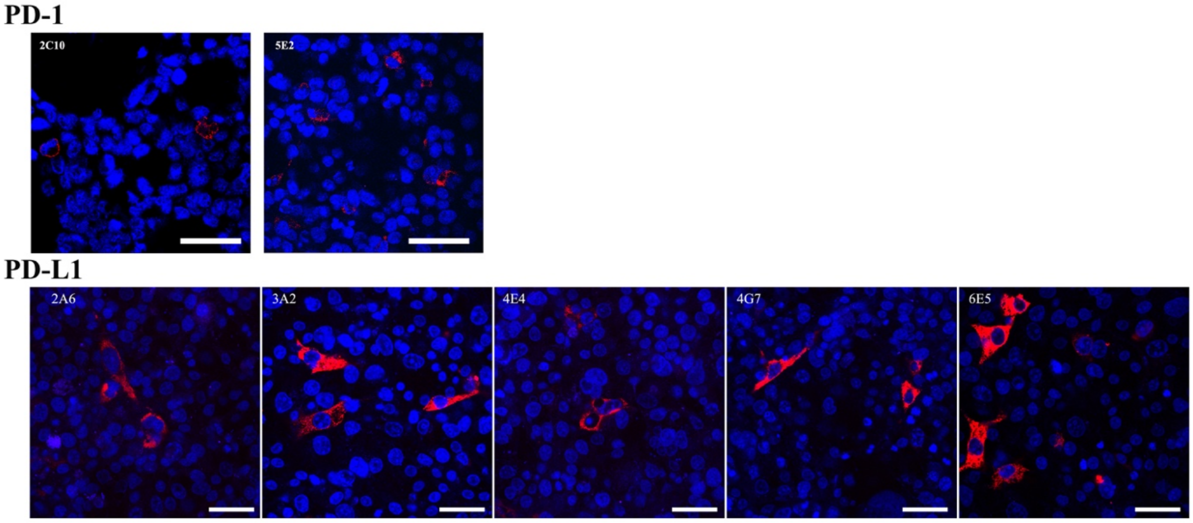

Supplement: Supplementary file 1 [file Data_Sheet_1.pdf]
